# A Teenager With Rash and Fever: Juvenile Systemic Lupus Erythematosus or Kawasaki Disease?

**DOI:** 10.3389/fped.2020.00149

**Published:** 2020-04-07

**Authors:** Marimar Saez-de-Ocariz, María José Pecero-Hidalgo, Francisco Rivas-Larrauri, Miguel García-Domínguez, Edna Venegas-Montoya, Martín Garrido-García, Marco Antonio Yamazaki-Nakashimada

**Affiliations:** ^1^Department of Dermatology, Instituto Nacional de Pediatría, Mexico City, Mexico; ^2^Department of Pediatrics, Instituto Nacional de Pediatría, Mexico City, Mexico; ^3^Department of Clinical Immunology, Instituto Nacional de Pediatría, Mexico City, Mexico; ^4^Department of Cardiology, Instituto Nacional de Pediatría, Mexico City, Mexico

**Keywords:** Kawasaki disease, juvenile systemic lupus erythematosus, intravenous immunoglobulins, adolescent, atypical Kawasaki disease

## Abstract

**Rationale:** Kawasaki disease (KD) is an acute vasculitis of small and medium vessels; whereas systemic lupus erythematosus (SLE) is a chronic systemic autoimmune disease. Their presentation is varied and not always straightforward, leading to misdiagnosis. There have been case reports of lupus onset mimicking KD and KD presenting as lupus-like. Coexistence of both diseases is also possible.

**Patient concerns:** We present three adolescents, one with fever, rash, arthritis, nephritis, lymphopenia, and coronary aneurysms, a second patient with rash, fever, aseptic meningitis, and seizures, and a third patient with fever, rash, and pleural effusion.

**Diagnoses:** The first patient was finally diagnosed with SLE and KD, the second patient initially diagnosed as KD but eventually SLE and the third patient was diagnosed at onset as lupus but finally diagnosed as KD.

**Interventions:** The first patient was treated with IVIG, corticosteroids, aspirin, coumadin and mycophenolate mofetil. The second patient was treated with IVIG, corticosteroids and methotrexate and the third patient with IVIG, aspirin and corticosteroids.

**Lessons:** Both diseases may mimic each other's clinical presentation. KD in adolescence presents with atypical signs, incomplete presentation, and develop coronary complications more commonly. An adolescent with fever and rash should include KD and SLE in the differential diagnosis.

## Introduction

Kawasaki disease (KD) and systemic lupus erythematosus (SLE) are immune mediated diseases characterized by varied clinical features that may include vasculitis (1–3). Vasculitis in lupus is most commonly due to the local deposition of immune complexes, but some patients have an inflammatory vasculopathy in the absence of local immune complex deposition (3). SLE can present coronary arteritis with aneurysm formation (4). We present three patients with overlapping features of KD and SLE. All patients and/or parents provided informed consent for publication of the cases.

### Case 1

A 16-year-old-male presented with a history of fever, weakness, headache with photophobia, abdominal pain, vomiting, and axillar lymphadenopathy. On physical examination he had persistent fever, conjunctival injection, malar erythema, erythematous and cracked lips, bilateral parotid enlargement, cervical lymphadenopathy and a diffuse photosensitive rash. Sicca symptoms were not present. KD was diagnosed, and intravenous immunoglobulins were started at 2 g/kg in addition to aspirin. The echocardiogram was within normal limits. After treatment, he was afebrile for 24 h, after which he presented seizures and neurological deterioration. Cranial computed tomography revealed changes suggestive of aseptic meningitis. A skin biopsy demonstrated an atrophic epidermis, necrotic keratinocytes, hydropic degeneration of the basal layer, basal membrane thickening and periadnexal and perivascular lymphocytic infiltration. Anti-Ro and IgM anti-β2-glycoprotein-1 antibodies were positive, antinuclear antibodies were negative. The diagnosis of systemic lupus erythematosus was made based on the presence of seizures, malar rash, photosensitivity and, positive anti-β2-glycoprotein-1 and anti-Ro antibodies. Corticosteroids, hydroxychloroquine, and methotrexate were started. He has been followed for more than 2 years, and the corticosteroids have been tapered with good evolution.

### Case 2

A 12-year-old male was transferred to our hospital with the diagnosis of lupus. He had a history of 20 days of fever, arthralgias, alopecia, a thoracic and abdominal rash, vomiting, oral ulcers, pleural effusion, pancytopenia, lymphopenia, and positive antinuclear antibodies (1:320). On physical examination the patient had palmoplantar erythema with desquamation and perineal erythema. Work-up showed a complete blood cell count within normal limits, increased AST (70 UI/l), ALT (59 UI/l) and bilirrubin (total 8.1 mg/dl, direct 5.5 mg/dl), and negative anti-dsDNA and anti-Sm antibodies. The echocardiogram revealed cardiomegaly and pericardial effusion. The presence of fever, palmoplantar erythema with desquamation, perineal erythema, elevated transaminases, gallbladder hydrops and pericardial effusion led to a diagnosis of incomplete KD and intravenous immunoglobulins, aspirin and corticosteroids were administered. On follow up, cardiac and liver abnormalities resolved.

### Case 3

**Figure 1:**
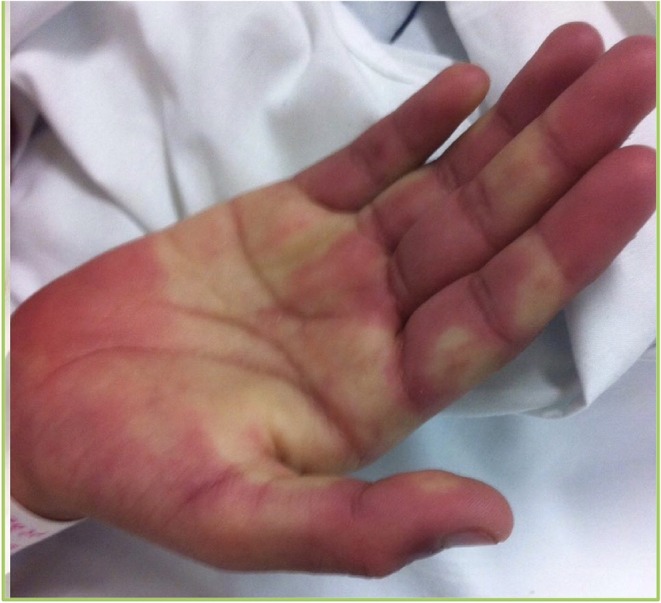
Erythema in palms accompanied by intense Raynaud's phenomenon.

**Figure 2:**
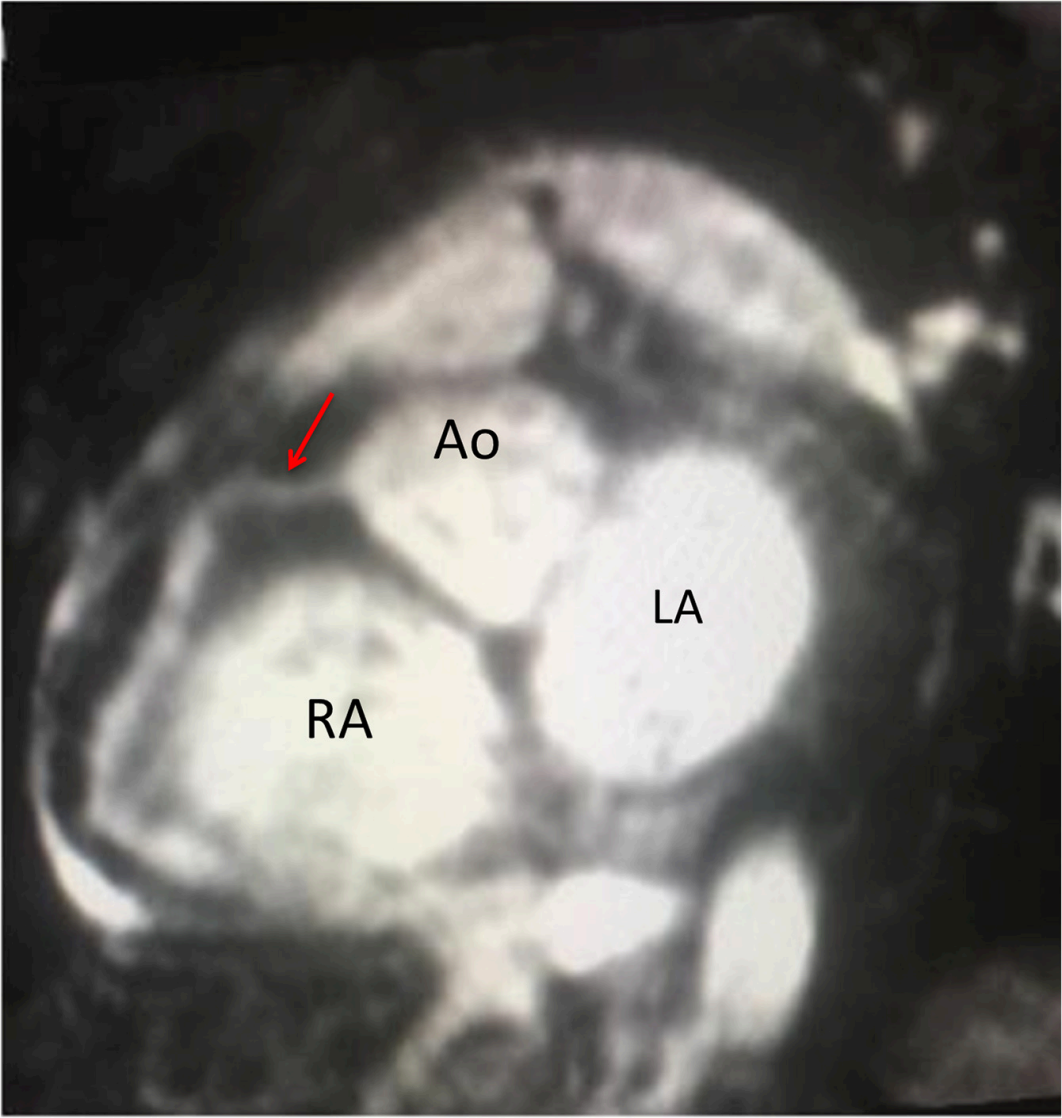
Magnetic resonance coronary angiography in a Whole-Heart iPAT sequence in a short axis view. Red Arrow: normal proximal right coronary artery 3 mm (z-score + 054), with dilated mid right coronary artery 6 mm (z-score + 7.35) and dilated distal right coronary artery 6 mm (z-score + 8.07). Ao, aorta; RA, Right atrium; LA, Left atrium (Courtesy of Dr. Roberto Cano).

An 11 year-old-female presented with a history of cervical adenopathy, followed 2 months later by left knee arthritis, malar rash, photosensitivity, dark urine and fever. On physical examination malar rash and intense Raynaud's phenomenon were noted (Figure 1). The diagnosis of lupus was made based on acute cutaneous lupus - malar erythema and photosensitivity -, arthritis, renal disease - cylindruria and proteinuria -, autoimmune hemolytic anemia, lymphopenia, positive antinuclear antibodies and anti-dsDNA antibodies. During her hospitalization fever continued and she presented erythematous crusted lips and a generalized rash with palmoplantar erythema. Intravenous immunoglobulins were administered with a presumptive diagnosis of Parvovirus-B19 infection. Methylprednisolone pulses were started, and improvement was observed. She was discharged with hydroxycloroquine, prednisone and mycophenolate mofetil. She presented periungueal desquamation while at home. One month later, she was readmitted to the hospital due to headache, seizures and persistent hypertension. Echocardiogram and heart MRI revealed large ectasia of the main left coronary artery (z-score + 6.12), large ectasia of the circumflex artery (z-score + 5.19), with normal proximal right coronary artery and large ectasia of the mid right coronary artery (z-score + 7.35) with mild mitral regurgitation (Figure 2).

## Discussion

Clinical criteria are used to diagnose KD with the presence of fever and principal clinical features involving the mouth, eyes, skin, hands and feet and cervical lymphadenopathy (Table 1). SLE is a complex autoimmune disease with variable clinical features. In the absence of SLE diagnostic criteria, SLE classification criteria are often used by clinicians to help identify some of the salient clinical features when making the diagnosis. Children who fulfill the ACR criteria, SLICC criteria or the new EULAR/ACR criteria are considered to have definitive SLE (Table 2). Of note is that in the recent EULAR/ACR criteria, fever is considered a criterion suggestive of SLE.

**Table 1:** Kawasaki disease classification criteria (AHA 2017 Guidelines).

Fever for at least 5 days in the presence of ≥ principal clinical features
Erythema and cracking of lips, strawberry tongue, and/or erythema of oral and pharingeal mucosa.
Bilateral bulbar conjunctival injection.
Erythema and edema of hands and feet in acute phase and/or peringueal desquamation in subacute phase.
Cervical lymphadenopathy ≥ 1.5 cm diameter.
A careful history may reveal that ≥1 principal clinical features were present during the illness but resolved by the time of presentation. Patients who lack full clinical features of classic KD are often evaluated for incomplete KD. If coronary artery abnormalities are detected, the diagnosis of KD is considered confirmed in most cases.
Other clinical findings: myocarditis, pericarditis, valvular regurgitation, gallbladder hydrops, aseptic meningitis, desquamating rash in the groin, anterior uveítis, erythema at the BCG inoculation site.

**Table 2:** Definitions of SLE classification criteria.

**ACR 1997**	**SLICC 2012**	**EULAR/ACR 2019**
	*Clinical criteria*	
1. Malar rash	1. Acute cutaneous lupus	1. Acute cutaneous lupus (malar rash or generalized maculopapular rash observed by a clinician)
2. Discoid rash	2. Chronic cutaneous lupus	2. Subacute cutaneous or discoid lupus
3. Photosensitivity		3. Fever
4. Oral or nasal ulcerations	3. Oral or nasopharyngeal ulcerations	4. Oral ulcers
	4. Nonscarring alopecia	5. Nonscarring alopecia
5. Nonerosive arthritis: Involving two or more joints, characterized by tenderness, swelling or effusion	5. Synovitis involving two or more joints	6. Joint involvement
6. Pleuritis or pericarditis	6. Serositis	7. Acute pericarditis
		8. Pleural or pericardial effusion
7. Renal disorders: persistent proteinuria or cellular casts	7. Renal disorders	9. Proteinuria >0.5 g/24 h
		10. Class II or V lupus nephritis on renal biopsy according to ISN/RPS 2003 classification
		11. Class III or IV lupus nephritis on renal biopsy according to international Society of Nephrology/Renal Pathology Society (ISN/RPS) 2003
8. Neurologic disorder: seizures or psychosis	8. Neurologic disorder	12. Delirium
		13. Psychosis
		14. Seizure
9. Hematologic disorders: • Hemolytic anemia or • Leukopenia or • Lymphopenia or • Thrombocytopenia	9. Hemolytic anemia	15. Leucopenia 16. Thrombocytopenia 17. Autoimmune hemolysis
	10. Leucopenia or lymphopenia	
10. Immunologic disorder: 1. anti-DNA antibody to native DNA or 2. anti-Sm antibody or 3. Positive antiphospholipid antibodies: 1) IgG or IgM anticardiolipin 2) positive lupus anticoagulant (LA) or 3) false positive test for syphilis	11. Thrombocytopenia	18. Antinuclear antibodies (ANA)
11. Positive antinuclear antibody by IFT or an equivalent assay	*Immunological criteria*	19. Low C3 OR low C4
	1. ANA level above laboratory reference range	
	2. Anti-dsANA antibody level above laboratory reference range	20. Low C3 AND low C4
	3. Anti-Sm antibody	
	4. Antiphospholipid antibody positive, by any of the following: -medium or high titer anti-cardiolipin -positive test for anti-beta-2glycoprotein	21. Anti-dsDNA antibodies OR anti-Smith (Sm) antibodies
	5. Low complement	
	6. Direct Coombs test in the absence of hemolytic anemia	22. Positive antiphospholipid antibodies

KD and SLE share several clinical manifestations: both diseases can present with fever, lymphadenopathy, arthritis or arthralgia, ocular and mucosal manifestations, rash and multisystemic involvement. However, the coexistence of both or misdiagnosis among them has seldom been reported (5–9). There are two previously reported cases of lupus-onset mimicking Kawasaki disease and vice versa and another three reported cases of the coexistence of both diseases (5–9) (Table 3).

**Table 3:** Cases with overlapping features of KD and SLE.

**References**	**Gender**	**Age**	**KD**	**SLE**		**Treatment**	**Final diagnosis**
Laxer et al. (5)	Female	10 m-5 yo	Fever (7 days), pruritic erythematous maculopapular rash, erythema of the palms and soles, bilateral noneudative conjunctivitis, rige posterior cervical lymph node, dry fissured lips, edema of her hands and feet., peeling of the skin over her fingers and toes	3 years later Fever, anorexia, photosensitivity, facial rash, livedo reticularis, painless palatal ulcer, generalized lymphadenopathy	Hemoglobin 8.3 g/dl, Leukocytes 4,000, ANA 1:640, Anti DNA positive, Rheumatoid Factor 40 UI (+). C3, C4 Markedly reduced. Immune Complexes 1,350 mcg/ml. Urianalysis: proteinuria and hematuria.	Aspirin 75 mg for 8 weeks. 3 years later. PDN 2 mg kg day.	KD and SLE
Marchetto et al. (7)	Male	15 yo	Fever, cheilitis, strawberry tongue, bilateral non exudative conjunctivitis with hemorrhages in the left eye and diffuse maculopapular rash, hands and feet with periungueal digital peeling	Butterfly rash on his face, arthralgia, muscle weakness, headache	ANA, antineurtrophil cytoplasmatic antibody, anti- DNA were negative. Positive anticardiolipin autoantibodies.	IVIG and acetyl salicylic acid. Recurrent KD Methylprednisolone an a second cycle of IVIG	KD
Diniz et al. (6)	Female	13 yo	Fever (7 days), bilateral bulbar nonexudative conjunctivitis, erythema of the oral an pharyngeal mucosa, cervical lymphadenopathy (2cc), erythema of Palms an diffuse maculopapular rash	Irritability, myalgia and arthritis (edema and tenderness in elbows and proximal interphalangeal joints in both hands an ankles),	Hemoglobin 9.7 gr/dl Urianalysis: Proteinuria 0.57 g/24 h. Leukocytes 3,000, Erythrocytes 1,000 Positive ANA 1:320, anti-dsDNA 516, anti-Ro. Negative anticardiolipin C3 42, C4 5	IVIG (2 g/kg do), and aspirin 80 mg/kg day Three pulses of intravenous methylprednisolone. PDN 30 mg/d Chloroquine Diphosphate, Azathioprine, aspirin 100 mg/d.	KD and SLE
Diniz et al. (6)	Female	4 yo	Fever (12 days), bilateral bulbar nonexudative conjunctivitis, cheilitis and strawberry tongue, cervidal lymphadenopathy (1.5cc), erythema of Palms, diffuse maculopapular rash, desquamation of the fingers and toes and in periungual region.	1 year later Irritability, Acute swelling of the eyelids, hands and feet, hypertension and pericarditis	Hemoglobin 7.4 g/dl, Leukocytes 3,800, Lymphocytes 874 Urinalysis: Leukocyturia Erythrocyturia Proteinuria g/24 h, C3 71 C4, <010 ANA 1:320 Anti-dsDNA 654.	IVIG (2 g/kgdo), and aspirin 80 mg/kg day 1 year later Three pulses of Intravenous methylprednisolone Cyclophosphamide Chloroquine Diphosphate	KD and SLE
Agarwal et al. (8)	Female	9 yo	Fever (Intermittent) Bilateral conjunctival erythema ECHO mild dilatation of the LMCA, and diffuse ectasia of the LAD, mild mitral regurgitation suggestive of carditis.	Abdominal pain arthralgias (ankles, wrists, right knee) weakness of lower extremities aphtous ulcer under the tongue	Hemoglobin 11.3 g/dL Leukocytes 3,100 ANA 1;2560 Positive Coombs Anti- dsDNA >200	Ethosuximide (discontinued) Intravenous Methylrednisolone pulse therapy (30 mg/kg day) for 3 days. Oral Steroids Methotrexate Hydroxychloroquine Aspirin (81 mg/day)	SLE
Agarwal et al. (8)	Female	6 yo	Fever Conjunctivitis non- exudative Cervical Adenopathy Rash 2 days later Recurrence of fever 2 day later Recurrence of Fever Sandpaper-like rash Cervical Lymphadenopathy ECHO dilated LMCA	Arthralgias (Ankle and Knee) Abdominal PAIN 4 days later Sinovitis of her wrists and knees.	Hemoglobin 9 g/dL ANA 1:640 Myeloperoxidase antibodies 28 mg/dL. 4 days later Hemoglobin 9.7 g/dL Platelet Count 530 k/ml Low C3 complement 64 mg/dL. Normal C4 complement ANA 1:2560 Myeloperoxidas and proteinase 3 antibodies negative. Antibodies-DSdna >200 Positive Combs Positive ENA-RNP	Intravenous Gammaglobulin 2 g/kg Aspirin 2 days later Intravenous Gammaglobulin 2 g/kg Aspirin 2 day later Intravenous Methylrednisolone pulse therapy (30 mg/kg day) for 3 days. Oral Steroids Hydroxicloroquine Aspirin 81 mg (daily) Methotrexate	SLE
Agarwal et al. (8)	Male (Family history for Lupus and Sarcoidosis)	13 yo	Eczema Fever (intermittent) Pruritic Rash Chill Bilaterally Injected Sclera Cervical Lymphadenopathy Bullous pemphigoid rash to the extremities Non pitting edema of lower extremities ECHO showed dilatation of the LMCA, LAD, and RCA without pericardial effusion, mild tricúspide insufficiency.	Joint pains Swelling of his hands and feet Palatal ulcers Synovitis of the small joints (hands, elbows, and knees)	Hemoglobin 4.9 g/dL. ANA 1:1280 Positive Coombs Antibodies-dsDNA >200 Positive anticardiolipin IgM, anti-Sm, anti-RNP, anti-SSA and SSB, β2-glycoprotein-1 antibodies. C3 20 mg/dL C4 < 2 mg/dL	Intravenous methylprednisolone pulse therapy (30 mg/kg day) for 3 days. Rituximab (750 mg/m^2^) on day 3 of steroid pulse, and a second dose given 2 weeks after Oral PDN Oral Enalapril Hydroxychloroquine Aspirin 81 mg/day 2 months later Mofetil mycophelolate	SLE
Argarwal et al. (8)	Female (Family history was notable for mother deceased due to complications of Rheumatoid Arthritis, SLE, Sjogren‘s syndrome, and dialysis'dependent end'stage renal disease).	13 yo	Fever Raynaud‘s phenomenon Bilateral pruritic red rash on her lower extremities Periorbital Edema ECHO demonstrated dilatation of the LMCA, LAD, and RCA, with perivascular echogenic brightness around the coronary branches. Borderline Leith ventricular hypertrophy and small circumferential pericardial effusion.	Headaches, swelling of both legs, bilateral synovitis of the elbows	Hemoglobin 6 g/dL BUN 33 mg/dL Cr 1.67 mg/dL Urinalysis hematuria an proteinuria >300 mg/dL. ANA 1:2560 Positive Coombs Antibodies-dsDNA >200 Positive RNP Positive anti-Sm antiRo antibodies. C3 17 mg/dL C4 2 mg/dL.	Intravenous Methylrednisolone pulse therapy (2 mg/kg day) for 3 days. Oral enalapril. Oral PDN Furosemide Hydroxychloroquine Mofetil mycophenolate	SLE
Zhang et al. (9)	Male	13 yo	Fever, rash, non-exudative conjunctivitis, cervical lymphadenopathy, arthralgia. ECHO showed coronary artery dilation (LCA 5.4 mm, RCA 6.9 mm)	Erythema, hepatosplenomegaly	Positive ANA and dsDNA antibodies. Hypocomplementemia. Positive Coombs. Leukopenia.	Intravenous methylprednisolone.	SLE (and KD?)
Case 1	Male	16 yo	Fever (1 month), painful cervical lymph nodes, rash on the trunk and extremities, conjunctival injection, cracked lips, oral mucosa erythematous	Malar erythema, Seizures and deterioration of neurological, Aseptic meningitis	Positive.β2-Anti- Glycoprotein-1 IgM type 44.02. anti Ro (+) antibodies.	IVIG (2 g/kgdo), and aspirin 80 mg/kg day later Methotrexate Hydroxychloroquine 400 mg/day. PDN 10 mg/day. Acenocumarine 2 mg/day	SLE
Case 2	Male	12 yo	Fever Palmoplantar erythema, desquamation hands and feet Perineal erythema, Gallbladder hydrops	Pleural and pericardial effusions, oral ulcers	Pancytopenia, Positive ANA	Methylprednisolone pulses IVIG	KD
Case 3	Female	11 yo	Fever, generalized rash, cervical lymphadenopathy, palmoplantar erythema, erythematous lips, desquamation hands	Malar rash, Raynaud's phenomenon, livedo reticularis	Positive ANA, anti-dsDNA, anti-Ro, anti-β2-glycoprotein-1, proteinuria Coombs positive hemolytic anemia	Methylprednisolone pulsesIVIGMofetil mycophenolate	SLE and KD

*IVIG, intravenous immunoglobulins; PDN, Prednisone; ANA, antinuclear antibodies*.

*ECHO, LMCA, Left main coronary artery; LAD, proximal left Anterior descending coronary artery; RCA, proximal right coronary arteria*.

The first patient was diagnosed with SLE and KD in an almost concurrent presentation, since she presented diagnostic criteria for both diseases. It can be discussed whether this case could only correspond to lupus with carditis, as the ones reported by Agarwal et al., however it is important to note that none of the four patients described by this author completed diagnostic criteria for KD (8). Recently, Zhang et al. (9) report a 13 year-old male who presented fever, rash, non-exudative conjunctivitis with cervical lymphadenopathy and an echocardiogram presenting coronary artery dilation. He was eventually diagnosed as SLE since he presented autoimmune hemolytic anemia, positive ANA, dsDNA and hypocomplementemia (9). As can be seen from previous reports (Table 3), both diseases can present simultaneously or with years of difference (5, 6, 9).

Coronary arteritis is not an exclusive feature of KD as other diseases like lupus and other vasculitis present this complication. In fact, coronary artery lesions have been documented in asymptomatic patients with microscopic polyangiitis, polyarteritis nodosa, and Wegener granulomatosis with MRI (10). Children with systemic onset juvenile idiopathic arthritis may present coronary artery dilation on echocardiograms similar to that observed for children with KD (11).

In our second patient the initial clinical picture made KD a diagnostic possibility; the skin biopsy was useful, as features were unequivocal for lupus. Parotitis was an unusual manifestation and can be present in both KD and lupus (12, 13). The third case was initially diagnosed as SLE, but eventually the clinical picture - despite atypical features such as pleural effusion, the response to treatment and the current health status under no medication, are more compatible with atypical KD (14).

Both KD and SLE share common features in terms of mechanisms of vascular inflammation and both may present with coronary artery dilatation. The two of them have been associated with the presence of anti-peroxiredoxin antibodies and the elevation of IL-17 (15, 16).

At this point, with the previously reported cases and our own it can be said that both diseases may mimic each other's clinical presentation. Interestingly, the majority of the patients that often present with the clinical challenge were tweens and teenagers (an unusual age for KD). KD in adolescence presents with atypical signs, incomplete presentation, and develop coronary complications more commonly (17). An adolescent with fever and rash should include KD and SLE in the differential diagnosis. As always in medicine, an accurate diagnosis is necessary to give appropriate treatment and reduce complications.

## Data Availability Statement

The raw data supporting the conclusions of this article will be made available by the authors, without undue reservation, to any qualified researcher.

## Ethics Statement

Signed informed consent was obtained from the parents and the patients.

## Author Contributions

MY-N, MS, and MP-H conceptualized and designed the study, reviewed, and revised the manuscript. MS and MY-N carried out the initial analyses and drafted the initial manuscript. FR-L and MG-G critically reviewed the manuscript. MP-H, EV-M, and MG-D recollected the data. All the authors were responsible for the treatment of the patient and read and approved the final manuscript.

### Conflict of Interest

MY-N has received lecture fees from Shire, CSL Behring and Octapharma. The remaining authors declare that the research was conducted in the absence of any commercial or financial relationships that could be construed as a potential conflict of interest.

## Abbreviations

KD: Kawasaki disease
SLE: systemic lupus erythematosus
IVIG: intravenous immunoglobulins
AST-Aspartate aminotransferase: ALT-Alanine transaminase.
